# Supplementary material for: Resolving the full spectrum of human genome variation using Linked-Reads
Source: Genome Res. 2019 Apr;29(4):635–45. doi: 10.1101/gr.234443.118 (PMC6442396; doi:10.1101/gr.234443.118)
Supplement: Supplemental Material [file supp_gr.234443.118_Supplemental_longranger-2.2.zip › longranger-2.2.tar.bz2!/lr2.2-public/lib/stan/cmdstan/stan/doc/api/html/agrad_2fwd_2functions_2log1p_8hpp_source.html]

Stan: src/stan/agrad/fwd/functions/log1p.hpp Source File


|  |  |
| --- | --- |
|  | Stan  2.5.0  probability, sampling & optimization |


- Main Page
- Related Pages
- Namespaces
- Classes
- Files

- File List
- File Members

All Classes Namespaces Files Functions Variables Typedefs Enumerations Enumerator Friends Macros Pages

- src
- stan
- agrad
- fwd
- functions

log1p.hpp

Go to the documentation of this file.

1 #ifndef STAN\_\_AGRAD\_\_FWD\_\_FUNCTIONS\_\_LOG1P\_HPP

2 #define STAN\_\_AGRAD\_\_FWD\_\_FUNCTIONS\_\_LOG1P\_HPP

3

4 #include <stan/agrad/fwd/fvar.hpp>

5 #include <stan/meta/traits.hpp>

6 #include <stan/math/functions/log1p.hpp>

7 #include <stan/math/constants.hpp>

8

9 namespace stan {

10

11  namespace agrad {

12

13  template <typename T>

14  inline

15  fvar<T>

16  log1p(const fvar<T>& x) {

17  using stan::math::log1p;

18  using stan::math::NOT\_A\_NUMBER;

19  if(x.val\_ < -1.0)

20  return fvar<T>(NOT\_A\_NUMBER, NOT\_A\_NUMBER);

21  else

22  return fvar<T>(log1p(x.val\_), x.d\_ / (1 + x.val\_));

23  }

24  }

25 }

26 #endif

stan::math::log1p

boost::math::tools::promote\_args< T >::type log1p(const T x)

Return the natural logarithm of one plus the specified value.

**Definition:** log1p.hpp:39

stan::math::NOT\_A\_NUMBER

const double NOT\_A\_NUMBER

(Quiet) not-a-number value.

**Definition:** constants.hpp:53

stan::agrad::fvar

**Definition:** fvar.hpp:13

traits.hpp

fvar.hpp

log1p.hpp

stan::agrad::fvar::d\_

T d\_

**Definition:** fvar.hpp:16

constants.hpp

stan::agrad::log1p

fvar< T > log1p(const fvar< T > &x)

**Definition:** log1p.hpp:16

stan::agrad::fvar::val\_

T val\_

**Definition:** fvar.hpp:15


---

[ Stan Home Page ]

*© 2011–2014,
Stan Development Team.*
